# From Bleeding Control to Health: Implementing Multidimensional and Multidisciplinary Hemophilia Care Into the Era of Therapeutic Innovation

**DOI:** 10.1111/hae.70376

**Published:** 2026-08-26

**Authors:** Nicolas Giraud, Yesim Dargaud

**Affiliations:** ^1^ Patient Representative French Hemophilia Association (AFH) Paris France; ^2^ French Reference Centre For Hemophilia Louis Pradel Hospital CHU de Lyon France; ^3^ UR4609 Hemostasis & Thrombosis Research Unit University Claude Bernard Lyon 1 Lyon France

**Keywords:** care model, hemophilia, normal life, rare inherited bleeding disorders

## Abstract

**Introduction:**

Over the past decade, hemophilia management has been transformed by modern prophylactic strategies, achieving dramatic reductions in annualized bleeding rates (ABR), often approaching zero. However, the conceptual evolution of hemophilia care toward multidimensional health assessment and comprehensive multidisciplinary management was initiated more than two decades ago through the adoption of functional, patient‐reported, and biopsychosocial outcome frameworks. Despite these advances, their implementation in routine clinical practice remains incomplete, highlighting a persistent disconnect between hemostatic control and overall health.

**Aim:**

To examine how recent therapeutic innovations should be integrated within established multidimensional models of hemophilia care and to identify the remaining barriers to their implementation.

**Methods:**

Narrative literature review combined with the joint perspective of a French patient representative and hematologist.

**Results:**

We discuss the need to operationalize multidimensional outcome assessment by integrating patient‐reported outcomes, joint health, functional status, participation, and sex‐specific bleeding phenotypes, including menstrual bleeding and limitations of ABR as a measure of treatment success. Therapeutic innovation also introduces new challenges requiring individualized care, behavioral adaptation, long‐term safety surveillance, and shared decision‐making. Persistent implementation gaps include fragmented care pathways, unequal access to multidisciplinary expertise and specialized laboratory support, and limited integration of psychological, functional, cardiovascular, and behavioral dimensions into routine care.

**Conclusions:**

The challenge for modern hemophilia care is no longer to redefine comprehensive, patient‐centered management, but to translate long‐established multidimensional frameworks into everyday clinical practice. Achieving sustained health, autonomy, and meaningful participation will require strengthening multidisciplinary care models, and ensuring equitable implementation of comprehensive care.

## 1 Introduction

Over the past decade, hemophilia management has undergone a groundbreaking therapeutic transformation, with annual bleeding rates (ABR) reaching historically low levels across hemophilia A and B. Phase III trials of extended half‐life (EHL) factor concentrates established that less frequent intravenous prophylaxis can maintain strong bleed protection: in pivotal studies of recombinant FVIII‐Fc and FIX‐Fc, individualized prophylaxis achieved median ABRs in the low single digits while reducing infusion burden compared with standard half‐life regimens [1, 2]. A further paradigm shift came with non‐factor therapies. In the HAVEN program, the bispecific antibody emicizumab delivered profound reductions in treated bleeding, including an ∼87% lower bleeding rate versus no prophylaxis in people with inhibitors (HAVEN 1) and near‐zero median treated ABR in people without inhibitors (HAVEN 3), demonstrating high efficacy irrespective of inhibitor status [3, 4]. Rebalancing therapies have consolidated this “inhibitor‐independent” era. The antithrombin‐lowering siRNA fitusiran significantly reduced ABR in Phase III trials across hemophilia A and B, with and without inhibitors, with a substantial proportion of participants experiencing zero treated bleeds [5]. Anti–tissue factor pathway inhibitor (TFPI) antibodies have demonstrated inhibitor‐independent efficacy: in Phase III trials, daily concizumab prophylaxis reduced ABR compared with no prophylaxis in patients both with and without inhibitors [6, 7], and similar findings were observed with once‐weekly marstacimab prophylaxis [8]. In parallel, gene therapy has expanded the paradigm of sustained bleeding protection in selected patients [9]. Long‐term follow‐up from pivotal trials demonstrates durable factor expression exceeding 10 years in hemophilia B and up to 5 years in hemophilia A. Across studies, ABR was markedly reduced compared with prior prophylaxis, allowing most participants to discontinue routine factor replacement [9]. Despite these remarkable therapeutic advances, can hemophilia truly be considered a solved problem?

Although the conceptual framework for comprehensive hemophilia care has been well established, its implementation through routine multidimensional assessment and integrated multidisciplinary care remains incomplete. Since the early 2000s, the World Health Organization's International Classification of Functioning, Disability and Health (ICF) has progressively provided the conceptual framework for hemophilia outcome assessment, extending evaluation beyond bleeding episodes to encompass body functions and structures, activities, participation, contextual factors, and patient‐reported outcomes [10]. In parallel, functional assessment tools such as the Functional Independence Score in Hemophilia (FISH) [11], the Hemophilia Activities List (HAL) [12, 13], the Canadian Occupational Performance Measure (COMP) [14], and disease‐specific quality‐of‐life instruments were developed to capture dimensions of health not reflected by bleeding rates alone. Subsequently, several clinical trials incorporated these functional outcome measures alongside traditional clinical and radiological joint assessment scores, recognizing the importance of evaluating patients' functional status in addition to structural joint outcomes [15]. Remarkably, although the ICF framework was introduced more than 25 years ago and functional assessment instruments and hemophilia‐specific quality‐of‐life measures have been available for nearly two decades, their translation into routine clinical practice has remained limited. Why has a framework that is universally accepted conceptually had so little impact on everyday therapeutic decision‐making? If modern therapies aim not only to prevent bleeding but to normalize life, then the relevant endpoint must extend beyond ABR to cover the broader reality of living with a rare bleeding disorder—patients’ sense of safety, freedom, and ability to live without the constant shadow of disease both now and in the long term.

## 2 Beyond ABR: Capturing What Truly Matters in Hemophilia

Historically, outcome assessment in hemophilia focused primarily on joint impairment and bleeding frequency, using clinical joint scores (e.g., Gilbert score) [16], radiological assessments (e.g., Pettersson score) [17], and ABR as the principal indicators of disease severity and treatment efficacy. The limitations of impairment‐based outcome measures progressively became apparent, as they failed to capture key dimensions of health, including functional capacity, participation, psychosocial well‐being, and patient‐defined treatment goals. Yet, despite broad international endorsement of multidimensional assessment, routine clinical practice and therapeutic decision‐making continue to rely predominantly on bleeding outcomes.

Despite the profound reduction in bleeding rates achieved with modern therapies, many adults with hemophilia—including younger adults—continue to live with the accumulated consequences of earlier disease. Established hemophilic arthropathy, chronic pain, reduced mobility, and functional asymmetry remain common, meaning that zero ABR does not equate to restored musculoskeletal health or full physical capacity [18, 19, 20]. For these patients, daily limitations are often driven less by the fear of new bleeds than by the enduring impact of prior joint damage [21, 22]. This reality invites a reconsideration of what is meant by a “normal life” in hemophilia. If contemporary therapies aim not only to suppress bleeding but to transform lived experience, the objective may need to shift from restoring a “normality” to optimizing long‐term function, autonomy, and well‐being for each individual [23] (Figure 1). In this context, outcomes that extend beyond traditional hemostatic metrics become essential. Patient‐reported outcomes (PROs) and quality‐of‐life measures address this gap by incorporating domains that traditional hemostatic metrics overlook, including pain burden, treatment convenience, participation in daily activities, psychological security, and perceived treatment freedom [24, 25]. Their strength lies in repositioning the patient's experience as a central measure of efficacy and in differentiating therapies with similar bleeding outcomes but distinct impacts on daily life [26, 27]. Yet PROs also present methodological challenges: heterogeneity of instruments, variable responsiveness, contextual influences, and the need for clearly defined thresholds of clinical relevance for both men and women [28, 29].

**FIGURE 1:**
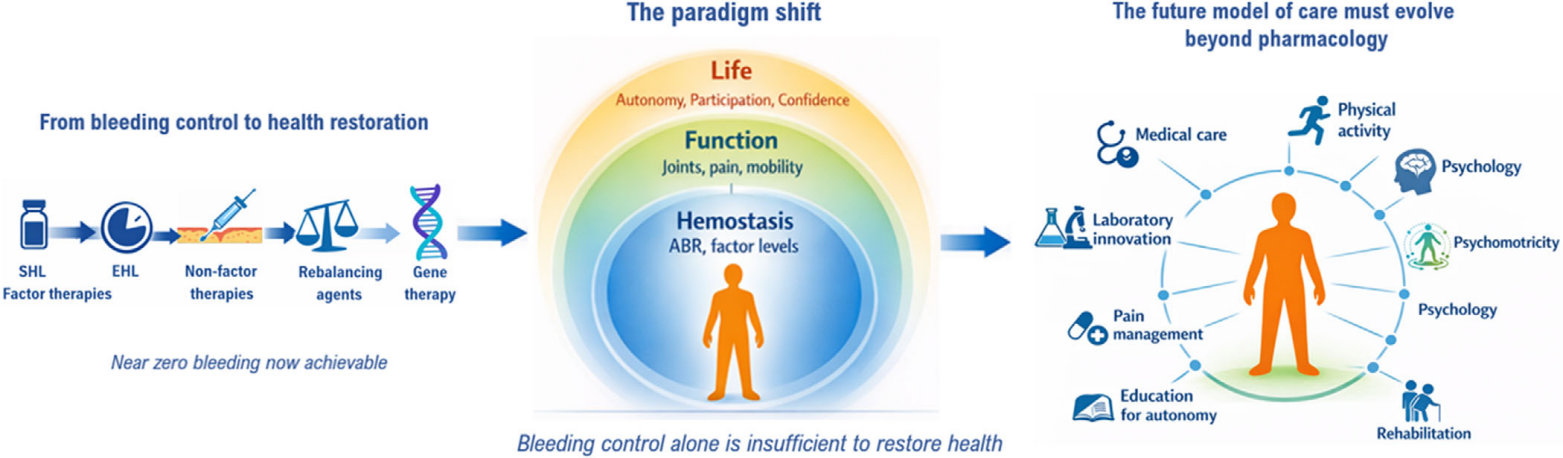
From bleeding control to health restoration in hemophilia care. Therapeutic innovations now enable near‐zero bleeding rates (left). However, improved hemostasis alone does not ensure full health restoration, which also depends on functional outcomes and life participation (center). The future model of care therefore places the patient within a multidisciplinary network integrating medical, rehabilitative, psychosocial, and laboratory approaches (right), emphasizing that optimal outcomes extend beyond pharmacological control toward comprehensive health restoration.

Joint outcomes represent a further underdeveloped dimension of current trial design. Structural and functional musculoskeletal changes evolve slowly and are difficult to capture within the timeframes of most pivotal studies, particularly in populations with pre‐existing damage [30]. Nevertheless, joint health, pain, and physical performance are often the primary determinants of long‐term disability and quality of life [31]. Imaging scores, functional assessments, and performance‐based measures therefore warrant greater prominence in long‐term evaluations as key indicators of disease modification and functional preservation.

Taken together, these considerations argue for a recalibration of outcome frameworks in hemophilia and other rare bleeding disorders. Future real‐life evaluations should adopt multidimensional endpoint strategies that integrate hemostatic control, patient‐centered outcomes, and functional or joint‐based measures. Importantly, such approaches must also account for sex‐specific bleeding phenotypes, including the systematic monitoring of menstrual bleeding in women, which is insufficiently captured by conventional ABR metrics. Aligning evaluation frameworks with these dimensions is essential to meet the contemporary goal of care: not only preventing bleeding, but enabling sustained function, autonomy, and full participation in everyday life.

### 2.1 Therapeutic Progress Drives Behavioral Evolution

The transition from historical protection to modern exposure represents a major psychosocial inflection point in the life course of adults with hemophilia. For decades, management strategies emphasized activity restriction to minimize bleeding risk, shaping durable cognitive schemas in which movement was associated with danger [32]. The arrival of highly effective prophylaxis—particularly non‐factor therapies such as emicizumab—has profoundly altered this landscape by dramatically reducing spontaneous bleeding and enabling unprecedented functional stability [3, 4]. However, biological protection does not automatically translate into adaptive behavior. Some individuals retain entrenched avoidance patterns that contribute to deconditioning and reduced quality of life, whereas others interpret near‐complete bleed control as physiological normalization and underestimate the vulnerability of previously damaged joints [33]. In the non‐factor era, this divergence may be accentuated by the simplicity of treatment administration and the perception of continuous protection, which can delay recognition of atypical joint pain or traumatic bleeding events [34]. Consequently, behavioral recalibration must be considered a core component of modern hemophilia care. Structured return‐to‐activity programs, education on symptom interpretation, and psychologically informed counseling are essential to align patient behavior with the realities of residual risk [35, 36]. Without this integrative approach, the therapeutic gains of transformative prophylaxis may coexist with preventable musculoskeletal deterioration or delayed clinical intervention.

### 2.2 Closing the Gap between Therapeutic Innovation and Comprehensive Hemophilia Care

Comprehensive multidisciplinary hemophilia care has long been recognized as the international standard and remains the cornerstone of optimal management. However, the rapid evolution of therapeutic options has exposed important implementation gaps between established care principles and routine clinical practice. Rather than redefining comprehensive care, the challenge now is to modernize its implementation. The multidimensional model of hemophilia care is well established; what is needed is its adaptation to a rapidly evolving therapeutic landscape. Multidisciplinary care models must evolve to support individualized treatment strategies, behavioral adaptation to highly effective prophylaxis, healthy ageing, cardiovascular risk management, increasingly sophisticated laboratory diagnostics, equitable access to specialized expertise, and genuine patient partnership. Only through this transformation can therapeutic innovation be translated into sustained health, functional independence, and meaningful improvements in quality of life.

The ageing of the hemophilia population illustrates this paradigm shift. Hypertension, obesity, diabetes, dyslipidemia, chronic kidney disease, and other cardiometabolic comorbidities are becoming increasingly prevalent, making thrombotic risk assessment an essential component of individualized care rather than a theoretical concern [37, 38]. For many patients, the central challenge is no longer simply preventing bleeding, but balancing effective hemostatic protection with cardiovascular health, thrombotic risk, and the uncertainties associated with lifelong therapies. Comprehensive care must therefore extend beyond traditional hemophilia‐related outcomes to incorporate systematic evaluation and management of age‐related comorbidities, particularly cardiovascular risk. Meeting these increasingly complex needs requires a fundamental shift from fragmented, specialty‐based care toward integrated multidisciplinary pathways. Rather than multiplying isolated consultations, hemophilia care should be organized around coordinated multidisciplinary clinics, ideally delivered within dedicated day‐hospital settings where patients can receive comprehensive assessment during a single visit. Collaboration between hematologists and other specialists should be tailored to patients' needs—for example, with cardiologists for cardiovascular disease, hepatologists for liver assessment, rheumatologists for arthropathy, and dentists for oral care—thereby reducing fragmentation of the care pathway and improving overall health management. Beyond improving clinical outcomes, this integrated model reduces fragmentation of the care pathway, limits conflicting recommendations, enhances therapeutic adherence, and refocuses care on the patient's overall health rather than bleeding control alone.

As pharmacological innovation has dramatically improved bleeding control, hemophilia has evolved from a disease defined primarily by acute bleeding into a chronic condition in which long‐term function, participation, and quality of life have become the principal determinants of health. This transformation calls for a corresponding evolution of comprehensive care centers beyond traditional biomedical expertise. New professional roles are essential to address health needs that extend beyond hemostatic control and cannot be met by pharmacological innovation alone. Psychologists with expertise in rare bleeding disorders, alongside patient partners trained in therapeutic education, can play a critical role in supporting treatment adherence, risk appraisal, and the management of anxiety related to pain, movement, or medicalization. They are also central in helping patients and families navigate the long‐term behavioral demands of prophylaxis and self‐management (Figure 2).

**FIGURE 2:**
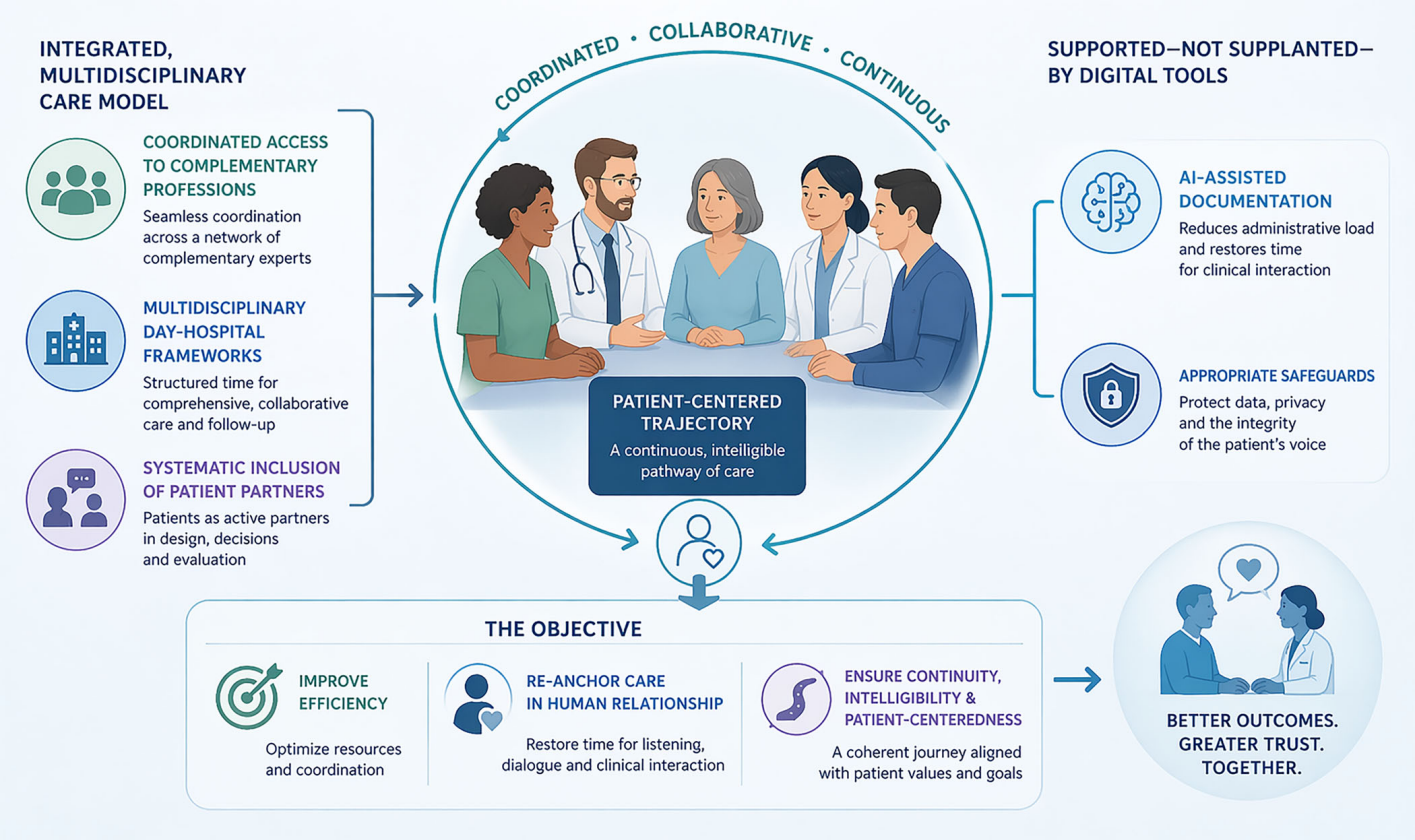
Decisive shift toward integrated multidisciplinary care for a patient‐centered trajectory.

At the same time, the functional landscape of hemophilia is changing across generations. Patients who grew up with repeated joint bleeds often required sustained physiotherapy to cope with established arthropathy and mobility limitations, making physiotherapists historically indispensable members of hemophilia centers [39]. In contrast, younger patients raised on modern prophylactic regimens that normalize, or nearly normalize, coagulation capacity will increasingly face different challenges. For them, the priority may shift from compensating for damage to safely developing physical capacity. This evolution implies a growing need for professionals specialized in adapted physical activity and sports coaching, who can guide patients toward confident, structured engagement in exercise and help overcome persistent avoidance behaviors inherited from earlier therapeutic eras. Physiotherapists will remain essential, but their role may become more targeted and episodic, focused on injury prevention, recovery, or specific musculoskeletal issues rather than lifelong compensation for joint disease [39], 40. Psychomotor therapists can assist in rebuilding motor confidence and proprioception, particularly in patients with longstanding joint disease. Chronic pain specialists and behavioral medicine experts are equally crucial, as pain persistence and maladaptive coping strategies often outlast bleeding risk [41].

True multidisciplinarity in hemophilia care is therefore no longer a supportive luxury but a structural requirement. The challenge today is not to redefine this model, but to strengthen and adapt it to the opportunities and complexities introduced by therapeutic innovation. Comprehensive hemophilia centers should continue to evolve as coordinated hubs that integrate hemostatic management with musculoskeletal health, rehabilitation, prevention, psychosocial support, behavioral adaptation, and long‐term management of ageing‐related comorbidities. This integrated approach is essential to ensure that therapeutic advances translate into meaningful improvements in physical function, autonomy, participation, and quality of life. Equally important is the development of structured regional and national networks linking comprehensive hemophilia centers with community hospitals, rehabilitation services, primary care providers, cardiology specialists, and specialized coagulation laboratories. Such collaborative models would promote equitable access to multidisciplinary expertise, facilitate standardized care pathways and laboratory support, and ensure that all patients benefit from comprehensive, patient‐centered care regardless of geographical location.

### 2.3 We Treat beyond What We Can Measure

The need to rethink hemophilia care is not limited to clinical teams; it extends equally to the hemostasis assays that underpin therapeutic decisions. Just as comprehensive centers must integrate new health professionals aligned with evolving patient needs, hematology laboratories must also adapt to the mechanistic shift introduced by non‐factor and rebalancing therapies [42, 43]. At present, a substantial gap persists between clinical innovation and biological assessment. Traditional factor level assays and aPTT‐based readouts do not reliably capture the global hemostatic balance induced by agents such as emicizumab, and assay interferences remain a practical challenge in routine laboratories [42]. Much of our understanding of “effective hemostasis” under these treatments still relies on indirect laboratory surrogates or extrapolations from animal models [44] that only partially reflect human physiology and clinical variability [45]. For patients living with congenital bleeding disorders, this creates an additional layer of uncertainty: therapeutic success may be assumed rather than objectively demonstrated. Closing this gap requires the development of validated global assays, clinically correlated biomarkers, and perioperative monitoring strategies capable of capturing true hemostatic competence in humans [42]. Without such tools, multidisciplinarity risks advancing on the clinical front while remaining biologically constrained, leaving clinicians and patients to navigate modern therapies with measurement systems rooted in a previous therapeutic paradigm.

Beyond assay development, maintaining access to specialized coagulation laboratories represents an increasing challenge. Current workforce policies in many tertiary hospital laboratories increasingly favor the pooling of laboratory technicians and engineers across multiple disciplines, including hemostasis, hematology, and clinical chemistry. Although operationally efficient, this model progressively dilutes disease‐specific technical expertise and threatens the sustainability of specialized coagulation laboratories. As laboratory diagnostics become increasingly sophisticated, preserving dedicated technical expertise will be as critical as developing new assays. Strengthening national laboratory networks and reinforcing collaboration between expert centers and peripheral hospitals will therefore become essential.

### 2.4 Living With Protection—Deciding With Uncertainty

The uncertainties surrounding biological monitoring extend far beyond the laboratory and shape the complexity of shared decision‐making in the era of novel hemophilia therapies. In the absence of validated tools capable of demonstrating both effective hemostatic protection and the absence of prothrombotic risk, therapeutic choices are frequently made within a framework of incomplete certainty [46, 47].

Non‐factor therapies, rebalancing strategies, and gene‐based approaches have shifted clinical decision‐making away from a singular focus on bleeding prevention toward a multidimensional evaluation of competing risks, encompassing long‐term safety, durability of response, and the potential for hypercoagulability or thrombotic complications in selected clinical settings.[3, 43] Although thrombotic events remain uncommon, experience with novel therapies has highlighted the need for continued vigilance, particularly in situations involving concomitant procoagulant treatments, perioperative management, or patients with pre‐existing cardiovascular risk factors.

Shared decision‐making, although intended to enhance autonomy, therefore introduces a substantial cognitive and emotional burden: patients must integrate uncertain evidence, anticipate long‐term consequences, and assume responsibility for choices whose hemostatic implications remain only partially measurable. In this context, achieving an annualized bleeding rate of zero cannot be regarded as a sufficient therapeutic endpoint; clinicians must also recognize and address the psychological weight generated by persistent uncertainty.

### 2.5 Redefining Normal Life across the Bleeding Disorders Spectrum

The notion of a “normal life” for people with hemophilia deserves critical redefinition in light of recent therapeutic progress. For decades, “normality” largely meant the absence of severe bleeding; today, it should be understood as sustained physical, psychological, and social participation with the lowest possible medical burden—an objective explicitly aligned with contemporary comprehensive care principles [20]. Yet this reframing remains unevenly distributed across the bleeding‐disorders community. The current wave of transformative, burden‐sparing innovation has predominantly benefited hemophilia, whereas many other rare inherited bleeding disorders still rely on low‐evidence, expert‐consensus‐based strategies and lack disorder‐specific options that simultaneously improve efficacy and reduce burden [48, 49]. For these patients—and for women and girls with bleeding disorders, who continue to face under‐recognition and diagnostic delay—the hemophilia experience should function as a shortcut rather than a precedent [50, 51]. The goal should not be for them to repeat the same incremental and difficult intermediate stages of progress, but to accelerate directly toward care pathways that couple effective hemostatic control with autonomy, quality of life, and sex‐specific and disorder‐specific needs.

## 3 Conclusion

Therapeutic innovation in hemophilia has transformed the clinical landscape, making sustained bleed prevention an achievable goal for many patients. Yet, normalization of hemostasis does not automatically translate into restored health or into a truly “hemophilia‐free mind” [52]. Long‐term outcomes now depend on preserving joint function, reducing pain, supporting psychosocial well‐being, and enabling full societal participation. The next phase of progress must therefore extend beyond pharmacological innovation to address a more structural limitation: the fragmentation of the care pathway. Highly specialized expertise and the multiplication of stakeholders, while essential, too often disperse clinical effort, dilute accountability, and ultimately constrain both patient adherence and meaningful clinical listening. In parallel, prevailing narratives that overemphasize therapeutic breakthroughs—whether framed as a “hemophilia‐free mind” or anchored in the promise of gene therapy—risk obscuring the lived reality of patients and diverting attention from the operational gaps that persist in 2026.

A decisive shift is needed toward integrated, multidisciplinary care models, structured around coordinated access to complementary professions, multidisciplinary day‐hospital frameworks, and the systematic inclusion of patient partners. Such models must be supported—not supplanted—by digital tools, including AI‐assisted documentation, designed to restore time for clinical interaction while preserving the integrity of the patient's voice through appropriate safeguards. The objective is not only to improve efficiency, but to re‐anchor care in a continuous, intelligible, and patient‐centered trajectory.

Reframing outcomes is central to this transformation. Moving beyond bleeding control alone toward health restoration, functional autonomy, and long‐term therapeutic education defines a more relevant and durable paradigm for rare inherited bleeding disorders. In this perspective, initiatives such as the Centre of Excellence on Patient and Public Partnership in Canada (https://ceppp.ca/) provide a conceptual and operational foundation for embedding partnership as a core scientific and organizational principle. The challenge now is to translate this vision into practice at scale, and to engage openly in the scientific debate required to shift the center of gravity of care toward integration, utility, and patient‐defined value.

## Author Contributions

Yesim Dargaud wrote the initial draft. Nicolas Giraud provided critical revisions and contributed the patient perspective. All authors approved the final version.

## Acknowlegments

Figures were generated using Open AI (Chat GPT Plus)

Open access publication funding provided by COUPERIN CY26.

## Funding

The authors have nothing to report.

## Conflicts of Interest

The authors declare no conflicts of interest related to this article.

## Data Availability

The data that support the findings of this manuscript are available upon request to the corresponding author
